# Study on the efficacy, safety, and biomarkers of nusinersen in type II and III spinal muscular atrophy in children

**DOI:** 10.3389/fped.2023.1294405

**Published:** 2023-12-04

**Authors:** Liyuan Chen, Fen Liu, Danna Fang, Jianwei Li

**Affiliations:** Department of Neurology, Dongguan Children’s Hospital Affiliated to Guangdong Medical University, Dongguan, China

**Keywords:** nusinersen, SMA, motor function, platelet, urinary protein

## Abstract

**Introduction/aims:**

The time span for the approval of nusinersen to treat SMA remains short. Most studies on the efficacy and safety of this drug within clinical trials, are lacking real-world research data. This study is based on real-world studies of SMA patients in children with type II and III SMA and is committed to objectively evaluating the effectiveness and safety of this drug.

**Methods:**

A retrospective analysis was conducted on the clinical data of 18 children with type II and III SMA from January 2022 to June 2023. The motor function assessment scale, SMN protein, platelet, liver and kidney function, and other laboratory indicators of all patients before and after treatment were collected for statistical analysis.

**Results:**

After load dose treatment (after 64 days of treatment), compared with baseline, the Revised Upper Limb Module (RULM) of SMA patients showed significant improvement (improvement rate: 44%), confirming the short-term effectiveness of the drug. The increase in cerebrospinal fluid SMN protein was greater in patients with significant improvement in motor function than in patients without improvement in motor function. Compared with baseline, there was no significant increase in AST and ALT levels in SMA patients, indicating that the drug had almost no effect on the liver. After each treatment, thrombocytopenia and partial urinary protein positivity may occur, but it could recover before the next treatment. This indicates that nusinersen is potentially harmful to platelet and renal function, although the effect is weak and reversible.

**Discussion:**

Nusinersen has shown good efficacy and overall safety, but platelets and urinary protein are still indicators that require long-term monitoring. The increase in cerebrospinal fluid SMN protein was greater in patients with significant improvement in motor function than in patients without improvement in motor function.

## 1. Introduction

Spinal muscular atrophy (SMA) is caused by the deletion or mutation of the survival gene of motor neuron 1 (SMN1), which leads to insufficient production of functional SMN protein, resulting in muscle weakness and atrophy. Intelligence and sensation are often not affected. SMN2, as a highly homeotic gene of SMN1, cannot fully compensate for the lack of SMN protein caused by the deletion of the SMN1 gene due to the difference of one nucleotide (1–3). The SMN2 exon exceeds exon 7 during splicing, and the generated Pre RNA lacks exon 7, producing mostly low functional truncated SMN protein (SMN2 △ 7), with only a few full-length SMN proteins. Based on the above mechanism, the copy number of SMN2 is an important influencing factor in determining the severity of the disease (4).

Nusinersen is a modified antisense oligonucleotide (ASO) designed to compensate for the defect in the SMN protein caused by chromosome 5q mutation. It targets the splicing of the SMN2 gene and binds to the specific sequence downstream of the intron of exon 7 of SMN2 to increase the mRNA transcript (SMN2 mRNA) containing exon 7, thus increasing the encoded functional SMN protein (5–7).

In clinical trials of multiple symptomatic or asymptomatic SMA patients, nusinersen was significantly found to improve the motor milestones of SMA patients, although the improvement in motor function was closely related to the patient's age, onset time and start time of treatment.

Multiple studies have confirmed the safety of nusinersen (7, 8). Nevertheless, pharmacokinetic studies have shown that nusinersen cannot cross the blood‒brain barrier and is mainly eliminated through slow metabolism in tissues (9). Drug metabolism is slow. Does it truly have no effect on all organs of the human body? Previous studies have confirmed platelet depletion and renal function impairment in SMA patients treated with nusinersen. However, due to the frequent increases of discomfort from blood sampling and urine testing, most studies mainly monitor platelet and urine protein during the maintenance phase. However, platelet and urine protein during the loading phases are more worthy of our attention.

The purpose of this study is to analyze the efficacy of type II and III SMA patients treated with nusinersen, grouping according to clinical efficacy, detecting the expression of SMN protein in patients with different effects. To evaluate adverse reactions after medication, monitor platelet and urinary protein indicators during the loading phases, and speculate the cutoff time for possible thrombocytopenia and urinary protein.

## 2. Patients and methods

### 2.1. Study design and participants

**Figure 1:**
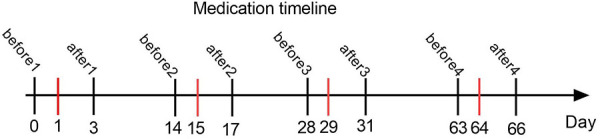
This graph shows the timeline of intrathecal injection of nusinersen and laboratory tests in all SMA patients. The red line represents the time of each injection of nusinersen. Before1, before2, before3, and before4 represent the days before the 1st, 2nd, 3rd, and 4th intrathecal injections of nusinersen, respectively, and after1, after2, after3, and after4 represent the days after the 1st, 2nd, 3rd, and 4th intrathecal injections of nusinersen, respectively.

This was a retrospective analysis of the clinical data of 18 patients with type II and III SMA admitted to our hospital from January 2022 to June 2023. All patients received their first treatment with nusinersen, and their baseline exercise levels were evaluated using the Revised Upper Limb Module (RULM), Hammersmith Functional Motor Scale Expanded (HFMSE), Six-minute walk test (6MWT) and Children's Hospital of Philadelphia Infant Test of Neuromuscular Disorders (CHOP INTEND) scores before treatment. Each patient selects one or more appropriate assessment methods based on their age and physical condition. RULM and HFMSE are used to evaluate patients who can sit or walk independently, CHOP INTEND is used to evaluate patients who cannot sit alone, and 6MWT is used to evaluate patients who can walk alone (10). Laboratory indicators such as platelets, urinary protein, and liver and kidney function were also measured. On Days 1, 15, 29, and 64, a dose of 12 mg of nusinersen was administered intravenously (Figure 1). The platelet, urinary protein, liver, and kidney function on the day before each treatment, as well as platelet and urinary protein levels on Day 2 after treatment, were recorded in detail. The motor function was reevaluated after 64 days of treatment (Figure 1). Collect 5 ml of cerebrospinal fluid before and 64 days after each intrathecal injection of nusinersen, and store it at −80°C for testing.

### 2.2. Reference values

The following reference values were used in this study: platelet count (100–300) × 10^9^/L; AST 0–40 U/L; ALT 0–40 U/L; Cr 24.9–69.7 µmol/L; UA 200–420 µmol/L.

### 2.3. Grouping method

18 patients were grouped based on all motor function assessment scales. Divided into valid the patients with any one or more evaluation results showing improvement in clinical symptoms are rated as the valid group, while the rest are classified as invalid group. The change in HFMSE score of ≥3 indicates improvement in clinical symptoms, the change in CHOP INTEND score of ≥4 indicates improvement in clinical symptoms, the change in RULM score of ≥2 indicates improvement in clinical symptoms, and for patients who can walk independently, the 6MWT result shows an increase in walking distance of ≥30 meters indicates improvement in clinical symptoms (10, 11). According to the above grouping, 8 patients (44%) were included in the valid group and 10 patients were included in the invalid group. Two groups of patients were tested for SMN protein in cerebrospinal fluid before and 64 days after treatment, respectively.

### 2.4. SMN protein in CSF

To assess SMN in CSF, we used an enzyme-linked immunosorbent assay (ELISA)(SED498Hu, 96T, distributed by CLOUD-CLONECORP. Wuhan, China).

The sample was not diluted and tested with the original solution. The sample detection range is 0.156 ng/ml–10 ng/ml. The minimum detection limit is 0.055 ng/ml.

### 2.5. Statistical methods

SPSS 27.0 statistical software was used for data analysis. Continuous variable data conforming to a normal distribution are expressed as the mean ± standard deviation (`x ± s) and were analyzed using an independent sample *t* test. Nonnormally distributed data are expressed as the median and interquartile spacing [*M* (*P*25, *P*75)], and a paired sample rank sum test (Wilcoxon rank sum test) was used.

## 3. Results

### 3.1. Clinical characteristics

A total of 22 pediatric SMA patients in this study received nusinersen treatment, including 15 cases of type II SMA and 7 cases of type III SMA. However, because the 4 patients did not receive at least 4 consecutive intrathecal injections of nusinersen, they were not considered in the data analysis. The detailed clinical characteristics of patients with different subtypes are shown in Table 1.

**Table 1:** Clinical characteristics of the study subjects.

Characteristics		SMA type II (*n* = 12)	SMA type III (*n* = 6)	Total (*n* = 18)
Gender	Male, *n* (%)	7 (58%)	3 (50%)	10 (56%)
	Female, *n* (%)	5 (42%)	3 (50%)	8 (44%)
Age at symptom onset (month) Mean ± SD (range)		9 ± 2.33 (6–12)	34 ± 36.72 (12–108)	17.33 ± 23.39 (6–108)
Age at treatment (year) Mean ± SD (range)		9 ± 4.32 (3–17)	11.5 ± 3.01 (7–15)	9.8 ± 4.02 (3–17)
SMN2 copy number	3	11 (92%)	4 (67%)	15 (83%)
	4	1 (8%)	2 (33%)	3 (17%)
Walk alone	Yes	0 (0%)	3 (50%)	3 (17%)
	No	12 (100%)	3 (50%)	15 (83%)
COBB angle, Mean ± SD (range)		31.8 ± 25.32 (0–83)	20.61 ± 16.18 (2–49)	28.09 ± 22.83 (0–83)

Ten SMA patients were male, and 8 were female. Fifteen patients had 3 SMN2 copies, and 3 patients had 4 SMN2 copies. Only 3 out of 18 patients were able to walk alone. The average age at symptom onset of all SMA patients was 17.33 ± 23.39 (6–108) months, and the average age at treatment was 9.8 ± 4.02 (3–17) years. Among them, the average onset age and treatment age of type II patients are earlier than those of type III patients. The average COBB angle of all SMA patients was 28.09 (0–83), and the COBB angle of type II patients was greater than that of type III patients.

### 3.2. Motor function assessment

After 64 days of treatment, the RULM score of SMA patients significantly increased (44%) compared to baseline (*P* = 0.041), and the HFMSE score also showed a slight increase (16%) compared to baseline, while the CHOP score slightly decreased compared to baseline. Two independently walking patients completed 6MWT, and their test results were (283 meters before treatment, 325 meters after treatment) and (256 meters before treatment, 269 meters after treatment) (Table 2).

**Table 2:** Comparison of RULM, HFMSE and CHOP between the study subjects before and 64 days after treatment.

Motor function assessment	Before the treatment	64 days after treatment	*Z* value	*P-*value
RULM (*n* = 18)	13 (7.75, 29)	18.5 (9, 28.75)	−2.048	0.041
HFMSE (*n* = 12)	14 (10.5, 44.75)	17 (11.25, 43.75)	−1.362	0.173
CHOP (*n* = 7)	42 (40, 43)	36 (31, 41)	−1.581	0.114

### 3.3. Expression levels of SMN protein in CSF of valid and invalid groups

**Figure 2:**
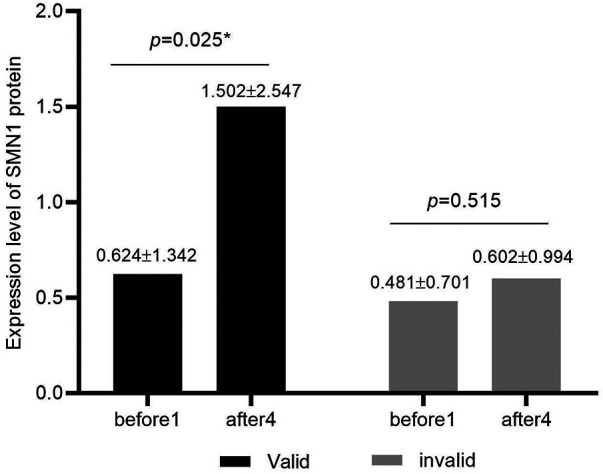
Expression levels of SMN protein in CSF of valid and invalid groups. Black represents the valid group (*n* = 8), while gray represents the invalid group (*n* = 12). The valid group showed significantly higher levels of SMN protein in cerebrospinal fluid after treatment with nusinersen compared to baseline (*p* = 0.025), while the invalid group showed no significant changes in SMN protein in cerebrospinal fluid after treatment with nusinersen compared to baseline (*p* = 0.515).

In the valid group, the SMN protein concentration values (mean and SD) before treatment and 64 days after treatment were 0.624 ± 1.342 and 1.502 ± 2.547, respectively. In the invalid group, the SMN protein concentration values (mean and SD) before treatment and 64 days after treatment were 0.481 ± 0.701 and 0.602 ± 0.994, respectively. The concentration of SMN in CSF of patients in the valid group showed a significant upward trend after treatment with nusinersen (*p* = 0.025), while the concentration of SMN in CSF of patients in the invalid group showed a slight upward trend after treatment with nusinersen, but the difference was not statistically significant (*p* = 0.515) (Figure 2).

### 3.4. Laboratory indicators (AST, ALT, Cr, UA)

**Figure 3:**
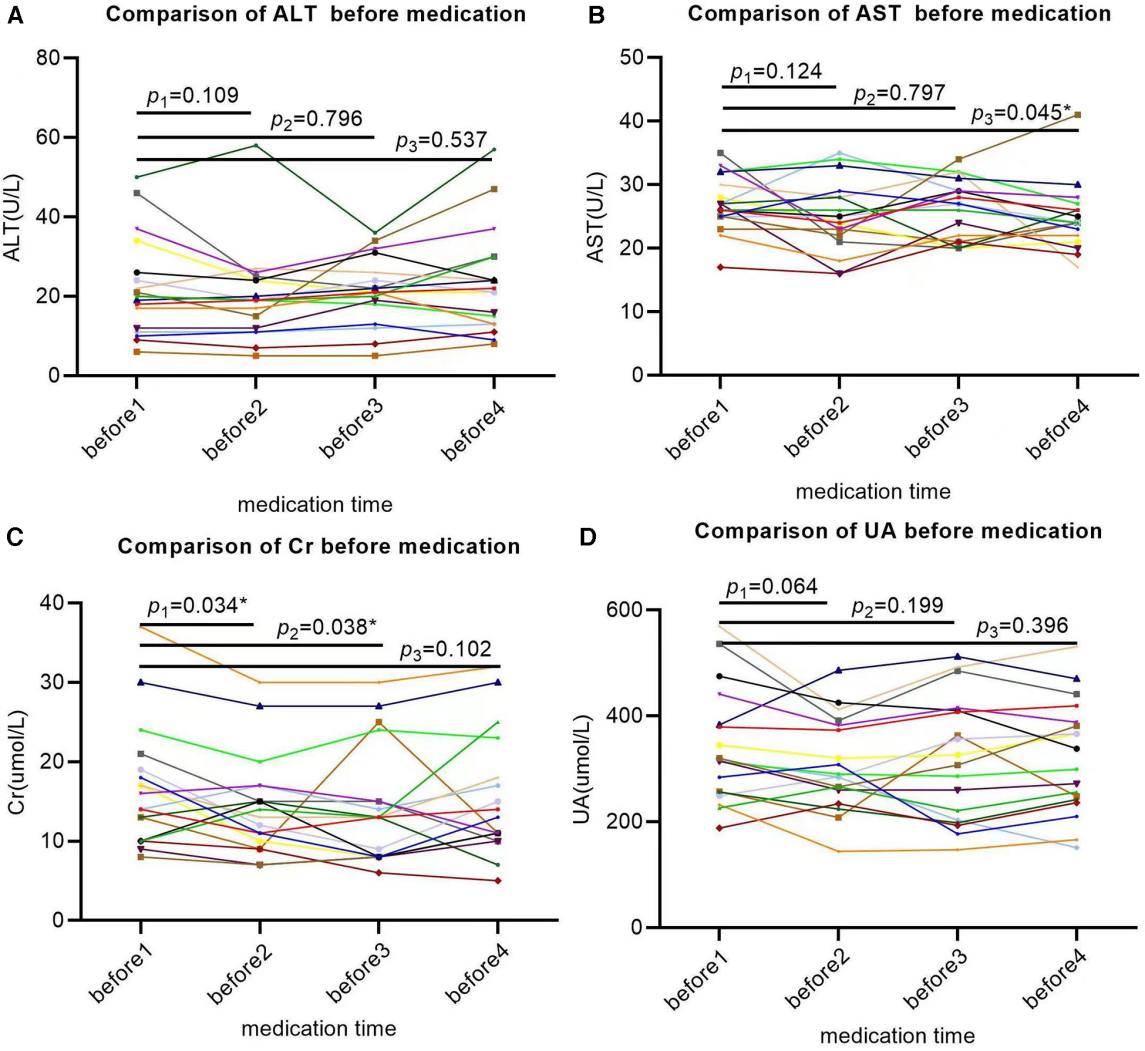
Changes in laboratory indicators (AST, ALT, Cr, UA) in SMA patients after 64 days of treatment. All X-axes represent different time points, while the Y-axis represents the values of each laboratory indicator. Each patient's laboratory indicator is represented by a separate colored line; (**A**) the changes in ALT were not significant at 14, 28, and 63 days after treatment; (**B**) AST showed no significant changes at 14 and 28 days after treatment but decreased significantly at 63 days after treatment; (**C**) Cr significantly decreased at 14 and 28 days after treatment but did not show significant changes at 63 days after treatment; (**D**) the changes in UA were not significant on the 14th, 28th, and 63rd days after treatment. * Indicates that the *P* value is less than 0.05.

The baseline ALT levels of SMA patients were basically within the normal range, except for two patients with mild elevation, and there was no significant change in ALT compared to the baseline levels after three rounds of treatment with nusinersen (*P*1 = 0.109, *P*2 = 0.796, *P*3 = 0.537; Figure 3A). The baseline level of AST was basically normal. After the first two rounds of treatment with nusinersen, there was no significant change in AST (*P*1 = 0.109, *P*2 = 0.796; Figure 3B), while after the third round of treatment with nusinersen, AST showed a slight decrease (*P*3 = 0.045; Figure 3B). Despite the decrease, AST remained within the normal range, except for one patient who had a slight increase. The baseline level of Cr generally decreased compared to normal values. After the first two rounds of treatment with nusinersen, Cr showed a slight decrease from baseline (*P*1 = 0.034, *P*2 = 0.038; Figure 3C), while after the third round of treatment with nusinersen, Cr showed no significant change from baseline (*P*3 = 0.102; Figure 3C). Four patients had a slight increase in baseline UA levels, while the remaining patients were basically normal. After three rounds of treatment with nusinersen, UA levels showed no significant changes compared to baseline levels (*P*1 = 0.064, *P*2 = 0.199, *P*3 = 0.396; Figure 3D).

### 3.5. Changes in platelets

**Figure 4:**
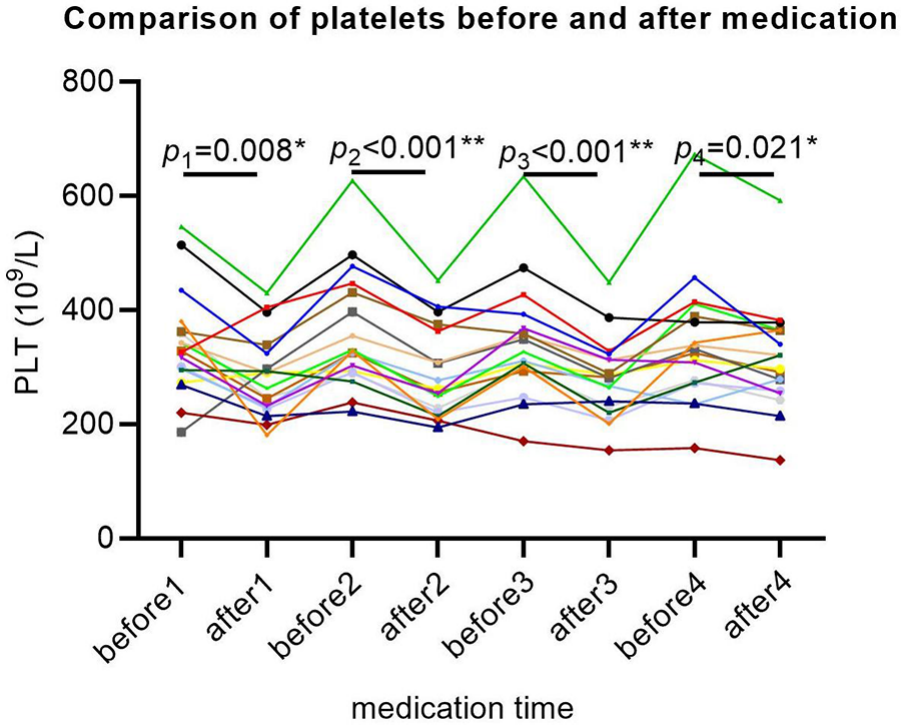
Platelet changes in SMA patients after 64 days of treatment. The *X*-axis represents different time points, the *Y*-axis represents platelet values, and each patient's platelet value is represented by a separate colored line. Each *P* value represents a significant difference in platelet levels between the first day before and two days after each medication. For example, *P*1 indicates a significant decrease in platelets 2 days after the first medication compared to the day before medication. Similarly, platelets are significantly reduced 2 days after each medication compared to the day before medication. * Indicates that the *P* value is less than 0.05, and ** indicates that the *P* value is less than 0.01.

The baseline platelet levels of all SMA patients were within the normal range. After each treatment with nusinersen, platelets showed a decreasing trend (*P*1 = 0.008, *P*2 < 0.001, *P*3 < 0.001, *P*4 = 0.021; Figure 4), and platelets rebounded again before the next treatment with nusinersen.

### 3.6. Changes in urine protein

**Figure 5:**
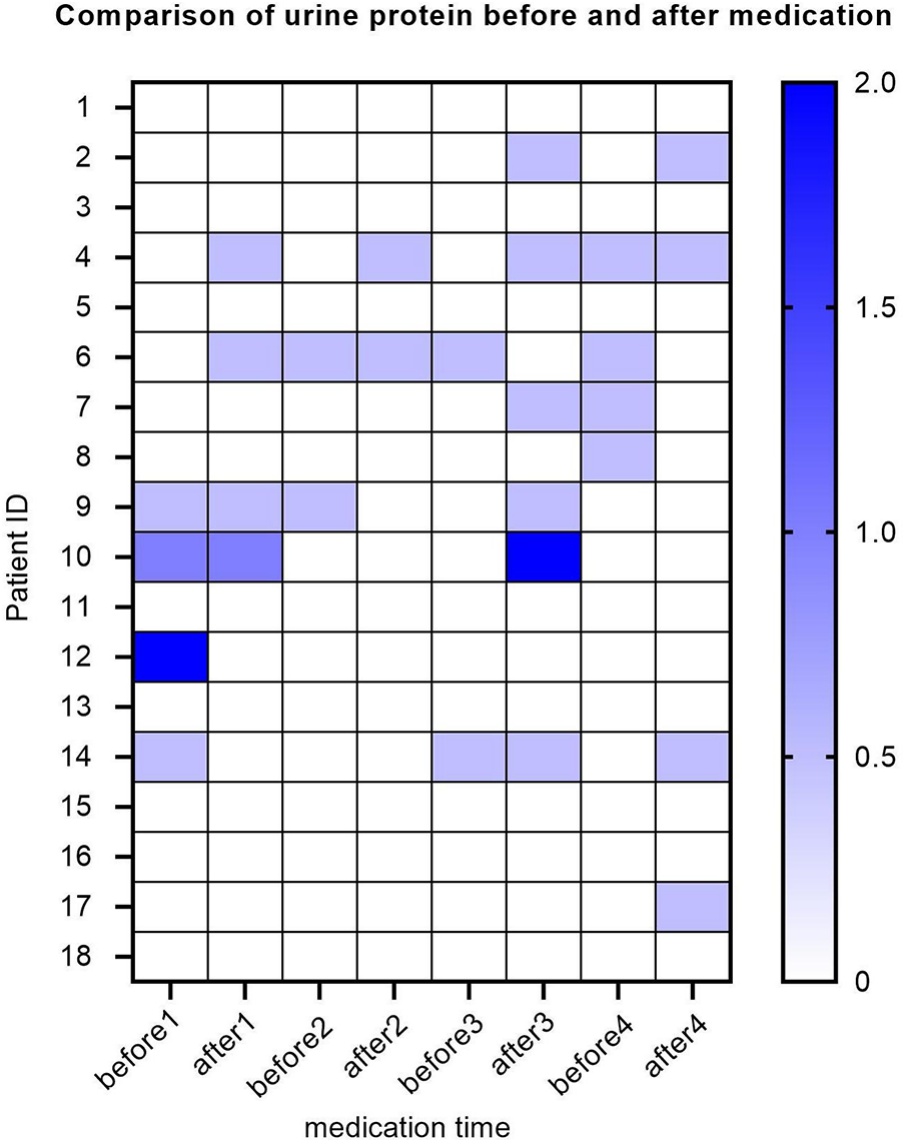
Heatmap of urinary protein levels for SMA patients. The *Y*-axis represents each patient, the *X*-axis represents different time points, and each small square represents the urine protein value of each patient at each time point. Here, different colors represent different urine protein levels, and the darker the color, the higher the urine protein value. Urinary protein is a qualitative value, with urinary protein (±) represented by a value of 0.5, urinary protein (+) represented by a value of 1, and urinary protein (++) represented by a value of 2.

Ten out of all SMA patients experience one or more episodes of elevated urinary protein levels. Four SMA patients had elevated baseline urinary protein levels, with two showing weak positivity (±), one showing positivity (+), and one showing strong positivity (++). The baseline urine protein levels of the remaining 14 patients were negative. After the first treatment with nusinersen, 2 patients turned negative for urinary protein, while 2 patients turned positive for urinary protein. After the second treatment with nusinersen, one patient's urine protein turned negative, and one patient's urine protein turned positive. After the third treatment with nusinersen, 1 patient's urine protein turned negative, and 5 patients' urine protein turned positive. After the fourth treatment with nusinersen, 3 patients had negative urinary protein levels, and 3 patients had positive urinary protein levels (Figure 5).

## 4. Discussion

The development and application of disease-modifying treatments have greatly changed the lives of SMA patients. The efficacy and safety of nusinersen have been confirmed in multiple clinical trials (5, 8, 12, 13). Due to the short duration of medication, some potential risks that may exist need to be recognized. In this article, we reviewed the research results of real-world data on 18 pediatric SMA patients treated with nusinersen, all of whom were diagnosed with type II and type III SMA. Our study is unique, as it provides detailed laboratory test results during the enhancement phase, including platelet and urinary protein levels. And we tested the expression of SMN protein in cerebrospinal fluid of patients with different effects.

Although the efficacy of nusinersen has been clinically confirmed, there are significant individual differences in the response of different SMA children to nusinersen drugs, which can be observed in our motor function score. Finding suitable biomarkers is of great significance for the treatment and prognosis evaluation of patients. The decline of SMN protein is an important mechanism for the occurrence of SMA diseases, and current specific therapeutic drugs aim to restore the expression of SMN protein. SMN protein is highly expressed in spinal cord and brain tissues, while it is low expressed in peripheral tissues (14). Detecting cerebrospinal fluid SMN protein can effectively evaluate the efficacy of this drug. Drug research has found that SMN protein levels in the brain and spinal cord of patients receiving treatment with nusinersen increase compared to the untreated control group (7). Our study compared the levels of SMN protein in patients with different therapeutic effects and found that patients with significant short-term efficacy showed a significant increase in SMN protein in the cerebrospinal fluid, while patients with poor efficacy did not show a significant increase in SMN protein in the cerebrospinal fluid. This result indicates that SMN protein can be used as a reliable biological indicator to evaluate the efficacy of nusinersen. In recent years, more and more scholars have found that neurofilament (NF) is another reliable biomarker (15, 16). The baseline levels of neurofilament light chains (NFL) in the plasma and cerebrospinal fluid of type I patients are higher than those of type 2 SMA patients. In type I and type II acute phase patients, NFL showed a rapid decline trend after load phase treatment with nusinersen, and then stabilized at lower levels. However, in type II chronic phase patients, NFL did not show significant changes (16). After several treatments with nusinersen, the level of NFL in the cerebrospinal fluid of SMA patients rapidly decreased, and there was a trend of higher levels with longer course of disease (17). Therefore, NFL can be used as an early response biomarker indicator for evaluating the efficacy of nusinersen. In addition, compound muscle action potential (CMAP) is the most concerned electrophysiological biomarker (15). Some studies have used it as a biomarker for evaluating the efficacy of SMA to evaluate the functional status of the motor unit pool in the body (18). Patients with faster CMAP increase tend to have better motor function after treatment with nusinersen (12). Therefore, CMAP may be a biomarker for monitoring the onset of symptoms in the disease, which will help determine the optimal treatment timing.

It is undeniable that many patients showed significant improvement in motor function after treatment with nusinersen. This can be confirmed by our RULM and HFMSE scores. Although our patients only received 4 doses of nusinersen treatment, the improvement in RULM scores was significant, and there was also a slight improvement in HFMSE scores. Our results are consistent with previous research results. A study showed that the RULM scores of SMA in children with types II and III continue to improve, especially in older and more severe phenotypes, where improvement in HFMSE scores may be limited due to scoliosis or contracture. The RULM score seems to be more inclined to monitor changes in motor function than in patients who cannot walk alone (19). There was no significant improvement in the CHOP score in our research results, which may be related to our shorter monitoring time and smaller number of cases.

Laboratory indicators are objective indicators for monitoring the side effects of new drugs. Past research data show that there are no serious complications, especially no damage to the liver. Continuous monitoring of transaminase (AST and ALT) is basically at a stable normal level (1). By monitoring AST and ALT after medication many times, our study did not find a trend of transaminase increase. Except for a few patients with slight elevation, most patients were basically at a normal level. The main metabolic pathway of nusinersen is the kidneys rather than the liver, so its impact on the liver is very weak, and our results also confirm this (20).

Renal toxicity is the main problem with nusinersen. Nagarajan et al. found that most patients had proteinuria before treatment, and this proteinuria characterization did not increase because of the use of drugs (21). But the specific mechanism is not yet clear. Our research results are similar. Four patients (22%) had proteinuria before treatment. We analyze the following possible reasons: none of our four patients are able to walk independently, have been in a wheelchair for a long time, and have a habit of holding urine for a long time. In order to reduce the frequency of urination, they may reduce the amount of water they drink, and these factors may all affect the damage to kidney function. In addition, during long-term management of these patients, we found that their urinary tract infection rate is also higher than that of normal individuals. There are individual patients with elevated urinary protein and elevated white blood cells before treatment, which is due to urinary tract infections. While after treatment, 1 patient had proteinuria that turned negative, and 3 patients still had discontinuous proteinuria. Comparing the prevalence of proteinuria between the sham-treated infants and the SMA patients before treatment, the results show that proteinuria is age dependent, which is due to the nature of SMA itself, rather than the side effects of nusinersen (22, 23). As an antisense oligonucleotide compound, nusinersen was chemically modified with phosphorothioate to improve the stability, absorption, and bioavailability of the drug. The modified compound is also the main source of toxicity, as it can bind low affinity serum albumin to avoid renal filtration. Although this mechanism can improve drug utilization, it may also cause drug accumulation in the proximal tubules and induce cell apoptosis to prevent the absorption of protein by the kidney (24). We found that after the third intrathecal injection of nusinersen, the number of proteinuria patients increased significantly. Due to the small sample size, it is still impossible to conclude whether the drug is related. Even if it is related to the drug, this side effect is reversible because we observed that most patients with positive proteinuria can basically turn negative before the next medication, which indicates that the amount of drug accumulation is controllable.

Creatinine and uric acid are also important monitoring indicators for renal dysfunction. After treatment, our patients did not show significant changes in uric acid levels, while creatinine showed a significant decrease after the first and second treatment with sodium loscinate. Interestingly, Stolte et al. also found this phenomenon in their study (25). Creatinine is a product of muscle tissue metabolism, while SMA patients themselves have reduced muscle content. In addition, due to a lack of exercise, muscle metabolism is relatively slow. The decrease in creatinine levels in SMA3 patients may also indicate that the disease is progressing (25). Some scholars have proposed that creatinine could be a candidate biomarker for disease progression (26, 27).

Both preclinical animal testing and clinical trials have reported that nusinersen may cause thrombocytopenia, but most of this reduction is mild and reversible, and this reduction is dose related (28). Therefore, it may be more important to monitor platelets at the load stage of treatment than at the maintenance stage (21). Nagarajan et al. found that on the 28th day of treatment with nusinersen, platelet reduction is the most obvious (21). We monitored the platelet changes before and 2 days after treatment during the loading phase and found that there was a decreasing trend in platelets after each injection of nusinersen. This phenomenon was observed in almost all patients, indicating that platelet decline was not an accidental event. However, before the next treatment, platelets rebounded, indicating that most thrombocytopenia occurs within a few days after treatment. Mild thrombocytopenia can be recovered within weeks or months, depending on the half-life of the drug (27). Our research results indicate that monitoring platelets before each treatment is necessary.

As a retrospective study, this study is limited to recording the motor function assessment scale and laboratory indicators during the patient's medical treatment process. Due to some patients’ physical discomfort or delayed treatment time, there are some missing data in the later stage. Therefore, the data in the maintenance stage were not included in this study. Small sample studies may have minimal results such as reduced accuracy and incompleteness. Future studies will include more samples and evaluate the potential side effects of the drug in a targeted manner to reduce patients’ economic costs and discomfort.

## 5. Conclusions

In this study, we evaluated the changes in motor function of patients before and after medication and found that the efficacy of nusinersen was initially observed during the load phase. And the patient's cerebrospinal fluid SMN protein was tested, and it was found that the increase in cerebrospinal fluid SMN protein was greater in patients with significant improvement in motor function than in patients without improvement in motor function. The analysis of laboratory indicators on the safety of the drug indicates that it is generally safe, with little impact on liver function. However, thrombocytopenia and urinary protein are still side effects that require our focus, and it is necessary to review them before each treatment.

## Data Availability

The raw data supporting the conclusions of this article will be made available by the authors, without undue reservation.
